# Spatial Heterogeneity, Host Movement and Mosquito-Borne Disease Transmission

**DOI:** 10.1371/journal.pone.0127552

**Published:** 2015-06-01

**Authors:** Miguel A. Acevedo, Olivia Prosper, Kenneth Lopiano, Nick Ruktanonchai, T. Trevor Caughlin, Maia Martcheva, Craig W. Osenberg, David L. Smith

**Affiliations:** 1 University of Puerto Rico–Río Piedras, Department of Biology, San Juan, PR, USA; 2 Dartmouth College, Department of Mathematics, Hanover, NH, USA; 3 Statistical and Applied Mathematical Sciences Institute, Durham, NC, USA; 4 University of Florida, Department of Biology, Gainesville, FL, USA; 5 University of Florida, Department of Mathematics, Gainesville, FL, USA; 6 University of Georgia, Odum School of Ecology, Athens, GA, USA; 7 Department of Epidemiology and Malaria Research Institute, John Hopkins Bloomberg School of Public Health, Baltimore, MD, USA; The University of Melbourne, AUSTRALIA

## Abstract

Mosquito-borne diseases are a global health priority disproportionately affecting low-income populations in tropical and sub-tropical countries. These pathogens live in mosquitoes and hosts that interact in spatially heterogeneous environments where hosts move between regions of varying transmission intensity. Although there is increasing interest in the implications of spatial processes for mosquito-borne disease dynamics, most of our understanding derives from models that assume spatially homogeneous transmission. Spatial variation in contact rates can influence transmission and the risk of epidemics, yet the interaction between spatial heterogeneity and movement of hosts remains relatively unexplored. Here we explore, analytically and through numerical simulations, how human mobility connects spatially heterogeneous mosquito populations, thereby influencing disease persistence (determined by the basic reproduction number *R*_0_), prevalence and their relationship. We show that, when local transmission rates are highly heterogeneous, *R*_0_ declines asymptotically as human mobility increases, but infection prevalence peaks at low to intermediate rates of movement and decreases asymptotically after this peak. Movement can reduce heterogeneity in exposure to mosquito biting. As a result, if biting intensity is high but uneven, infection prevalence increases with mobility despite reductions in *R*_0_. This increase in prevalence decreases with further increase in mobility because individuals do not spend enough time in high transmission patches, hence decreasing the number of new infections and overall prevalence. These results provide a better basis for understanding the interplay between spatial transmission heterogeneity and human mobility, and their combined influence on prevalence and *R*_0_.

## Introduction

More than half of the world’s population is infected with some kind of vector-borne pathogen [1–3], resulting in an enormous burden on human health, life, and economies [4]. Vector-borne diseases are most common in tropical and sub-tropical regions; however, their geographic distributions are shifting because of vector control, economic development, urbanization, climate change, land-use change, human mobility, and vector range expansion [5–9].

Mathematical models continue to play an important role in the scientific understanding of vector-borne disease dynamics and informing decisions regarding control [10–14] and elimination [15–17], owing to their ability to summarize complex spatio-temporal dynamics. Although there is increasing interest in the implications of spatial processes for vector-borne disease dynamics [18–22], most models that describe these dynamics assume spatially homogeneous transmission, and do not incorporate host movement [23–25]. Yet, heterogeneous transmission may be the rule in nature [26–28], where spatially heterogeneous transmission may arise due to spatial variation in vector habitat, vector control, temperature, and rainfall, influencing vector reproduction, vector survival and encounters between vectors and hosts [29, 30].

Movement of hosts among patches with different transmission rates links the pathogen transmission dynamics of these regions [31]. In the resulting disease transmission systems some patches may have environmental conditions that promote disease transmission and persistence (*i.e.*, hotspots), while other patches may not be able to sustain the disease without immigration of infectious hosts from hotspots [32]. Control strategies often focus on decreasing vectorial capacity in hotspots [33, 34] with some successes, such as malaria elimination from Puerto Rico [35], and some failures [36, 37], such as malaria control efforts in Burkina Faso [38]. An often overlooked factor when defining sites for control efforts is a patch’s connectivity to places of high transmission. For example, malaria cases during the 1998 outbreak in the city Pochutla, Mexico were likely caused by human movement into the city from nearby high transmission rural areas, despite active vector control in Pochutla [39]. Understanding the interaction between connectivity—defined by the rate of movement of hosts among patches—and spatial heterogeneity in transmission via mathematical models has the potential to better inform control and eradication strategies of mosquito-borne diseases in real-world settings [37, 40].

In this study, we ask, how host movement and spatial variation in transmission intensity influense malaria long-term persistence and prevalence. First, we show analytically that transmission intensity is an increasing function of spatial heterogeneity in a two-patch system, where the patches are connected by host movement. Second, we apply a multi-patch adaptation of the Ross-Macdonald modeling framework for malaria dynamics to explore the implications of spatial heterogeneity in transmission intensity and human movement for disease prevalence and persistence. The mosquitoes that transmit malaria typically move over much smaller spatial scales than their human hosts. Thus, we assume that mosquito populations are isolated in space. The varying size of mosquito populations across a landscape introduces spatial heterogeneity in transmission intensity. This heterogeneity, coupled with the fact that humans commonly move among areas with varying degrees of malaria transmission, makes malaria an ideal case study.

## Materials and Methods

The Ross-Macdonald modeling approach describes a set of simplifying assumptions that describe mosquito-borne disease transmission in terms of epidemiological and entomological processes [41]. Although it was originally developed to describe malaria dynamics, the modeling framework is simple enough to have broad applicability to other mosquito-borne infections. One of the most important contributions of the Ross-Macdonald model is the identification of the threshold parameter for invasion *R*_0_, or the basic reproductive number. Threshold quantities, such as *R*_0_, often form the basis of planning for malaria elimination. In some cases *R*_0_ also determines the long-term persistence of the infection. Here, we define persistence to mean *uniform strong persistence* of the disease; that is whether the disease will remain endemic in the population, and bounded below by some positive value, over the long term. Mathematically, a disease is *uniformly strongly persistent* if there exists some *ϵ* > 0 such that limsup_*t* → ∞_ *I*(*t*) ≥ *ϵ* for any *I*(0) > 0, where *I*(*t*) is the number of infected individuals at time *t* [42, 43].

To extend the Ross-Macdonald model to a landscape composed of *i* = 1, …, *Q* patches we need to account for the rate of immigration and emigration of humans among the *Q* patches. The full mathematical derivation of the multi-patch extension (Eq 1) from the original Ross-Macdonald model can be found in S1 Text.

For each patch *i*, the rates of change in the proportion of infected mosquitoes, the number of infected hosts, and the total number of humans are calculated as

$$\begin{array}{ccc} \frac{dz_{i}}{dt} & = & a_{i}c_{i}\frac{I_{i}}{N_{i}}\left(e^{-g_{i}n_{i}}-z_{i}\right)-g_{i}z_{i}\\ \frac{dI_{i}}{dt} & = & m_{i}a_{i}b_{i}z_{i}\left(N_{i}-I_{i}\right)-r_{i}I_{i}-I_{i}\sum_{j\neq i}^{Q}k_{ji}+\sum_{j\neq i}^{Q}k_{ij}I_{j}\\ \frac{dN_{i}}{dt} & = & -N_{i}\sum_{j\neq i}^{Q}k_{ji}+\sum_{j\neq i}^{Q}k_{ij}N_{j} \end{array}$$

where *N*_*i*_ describes the total size of the human population in patch *i*, *I*_*i*_ represents the number of infected hosts in patch *i*, *z*_*i*_ represents the proportion of infected mosquitoes in patch *i*, and *k*_*ji*_ represents the rate of movement of human hosts from patch *i* to patch *j*. Note that 1/*k*_*ji*_ describes the amount of time (days in this particular parameterization) an individual spends in patch *i* before moving to patch *j*. For simplicity, we assumed that the rate of host movement was symmetric between any two patches, and equal amongst all patches, such that *k* = *k*_*ij*_ = *k*_*ji*_. We further assumed that the initial human population densities for each patch were equal. This constraint on the initial condition, along with the assumption of symmetric movement, causes the population size of each patch to remain constant, that is, *dN*_*i*_/*dt* = 0 for all *i*. We also assumed that the only parameter that varies among patches is the ratio of mosquitoes to humans, *m*_*i*_. The rate *a*_*i*_ at which mosquitoes bite humans, the probability *c*_*i*_ a mosquito becomes infected given it has bitten an infected human, the probability *b*_*i*_ a susceptible human is infected given an infectious mosquito bite, the mosquito death rate *g*_*i*_, the human recovery rate *r*_*i*_, and the extrinsic incubation period (the incubation period for the parasite within the mosquito) *n*_*i*_, are all assumed constant across the landscape. Consequently, for all *i* = 1, …, *Q*, *a*_*i*_ = *a*, *b*_*i*_ = *b*, *c*_*i*_ = *c*, *g*_*i*_ = *g*, *r*_*i*_ = *r*, and *n*_*i*_ = *n*.

In this model there is no immunity conferred after infection. Furthermore, although host demography (births and deaths) can play an important role in transient disease dynamics, because our focus is the relationship between equilibrium prevalence and *R*_0_ under the assumption of constant patch population sizes, we omit host demography. Choosing constant birth rates Λ = *μN* and natural host mortality rates *μ* in each patch yields identical *R*_0_ and equilibria to our model, with the exception that *r* is replaced by *r* + *μ*. Thus, including host demography in this way would result in a slight decrease in *R*_0_ and prevalence by decreasing the infectious period. How host demography influences the relationship between *R*_0_ and prevalence when patch population sizes are not constant, and moreover, when host demography is heterogeneous, is an interesting question that remains to be explored. These simplifying assumptions yield the following system of 2*Q* equations,

$$\begin{array}{ccc} \frac{dz_{i}}{dt} & = & ac\frac{I_{i}}{N}\left(e^{-gn}-z_{i}\right)-gz_{i}\\ \frac{dI_{i}}{dt} & = & m_{i}abz_{i}\left(N-I_{i}\right)-rI_{i}-I_{i}\sum_{j\neq i}^{Q}k+\sum_{j\neq i}^{Q}kI_{j} \end{array}$$ (1)

### Analyses

Differences in the ratio of mosquitoes to humans, *m*_*i*_ results in a network of heterogeneous transmission, where each patch in the network is characterized by a different transmission intensity. The basic reproduction number for an isolated patch (i.e., one not connected to the network through human movement) is defined by $R_{0,i}=\frac{\alpha_{i}\beta }{rg}$, where *α*_*i*_: = *m*_*i*_ *abe* ^−*gn*^ and *β*: = *ac*, and is a measure of local transmission intensity. Furthermore, *R*_0,*i*_ is a threshold quantity determining whether disease will persist in patch *i* in the absence of connectivity. In particular, if *R*_0,*i*_ > 1, malaria will persist in patch *i*, while if *R*_0,*i*_ ≤ 1, it will go extinct in the absence of connectivity with other patches. *R*_0,*i*_ (local transmission) increases with the ratio of mosquitoes to humans *m*_*i*_, and if more transmission occurs, more people are infected at equilibrium. These results, however, do not necessarily hold in a network where hosts move among patches [20]. Indeed, movement can cause the disease to persist in a patch where it would otherwise die out [20, 44].

To address this limitation of the isolated patch reproduction number, we used the next generation approach [45, 46] to calculate *R*_0_ for the whole landscape. This approach requires the construction of a matrix *K* = *FV* ^−1^, where *J* = *F*−*V* is the Jacobian of the 2*Q*-dimensional system evaluated at the disease-free equilibrium, *F* is nonnegative, and *V* is a nonsingular M-matrix. *F* contains terms related to new infection events, and *V* contains terms of the Jacobian related to either recovery or migration events. This choice satisfies the conditions for the theory to hold, and the important consequence of this approach is that the spectral radius of the next generation matrix *ρ*(*K*) is less than one if and only if the disease-free equilibrium is locally asymptotically stable. Defining *R*_0_ = (*ρ*(*K*))^2^, we have that the disease-free equilibrium is locally asymptotically stable when *R*_0_ < 1 and unstable when *R*_0_ > 1. We proved (see S2 Text) that System (1) exhibits *uniform weak persistence* of the disease when *R*_0_ > 1; that is, when *R*_0_ > 1, there exists an *ϵ* > 0 such that ${\text{lim sup}}_{t\to \infty }\sum_{i=1}^{Q}I_{i}(t)+z_{i}(t)\geq \epsilon$, for any initial condition for which $\sum_{i=1}^{Q}I_{i}(0)+z_{i}(0)>0$. Furthermore, because our model is an autonomous ordinary differential equation, *uniform weak persistence* implies *uniform strong persistence*. Consequently, when *R*_0_ > 1, there exists an *ϵ* > 0 such that ${\text{lim inf}}_{t\to \infty }\sum_{i=1}^{Q}I_{i}(t)+z_{i}(t)\geq \epsilon$, for any initial condition for which $\sum_{i=1}^{Q}I_{i}(0)>0$[42, 43]. A generalization of our multi-patch system (see System (8) in [47]) exhibits a unique endemic equilibrium when *R*_0_ > 1 which is globally asymptotically stable. Likewise, the disease-free equilibrium for their model is globally asymptotically stable when *R*_0_ ≤ 1. In fact, Auger *et al.* [47] proved this result even when migration is neither constant across the landscape, nor symmetric.

Because *R*_0,*i*_ defines a threshold for disease persistence in an isolated patch and *R*_0_ defines a threshold for disease persistence in the connected network, we use these two quantities as surrogates for local patch persistence when patches are isolated, and persistence in the connected network as a whole, respectively. Prevalence, on the other hand, was calculated as the total proportion of infected hosts in the landscape at equilibrium.

Heterogeneity in transmission intensity was quantified using the coefficient of variation (CV) of the ratio of mosquito to humans (*m*) such that

$$\begin{array}{c} \text{CV}=\frac{s_{\bar{m}}}{\bar{m}}, \end{array}$$ (2)

where $\bar{m}$ describes the average ratio of mosquito to humans in the landscape and $s_{\bar{m}}$ represents the standard deviation associated with this average. This coefficient of variation is a simple measure commonly used in landscape ecology to quantify landscape heterogeneity [48].

We analyze two cases: (1) a simple two-patch system (*Q* = 2) where we study analytically the relationship between spatial heterogeneity, *R*_0_ and prevalence. Then, (2) we address a similar question in a multi-patch system (*Q* = 10) where each patch is characterized by their unique transmission intensity (see below).

### Two-patch analysis

We use an analytical approach (see S3 Text) to study the relationship between *R*_0_, prevalence, and spatial heterogeneity in the special case where the network is composed of two connected patches (*Q* = 2). Transmission heterogeneity in the system is created by choosing different values for *m*_1_ and *m*_2_, the ratio of mosquitoes to humans in the two patches, and quantified by the coefficient of variation, CV. We define $\bar{m}$ to be the average of *m*_1_ and *m*_2_, and study the behavior of *R*_0_ and prevalence as CV increases.

### Multi-patch simulation

**Fig 1:**
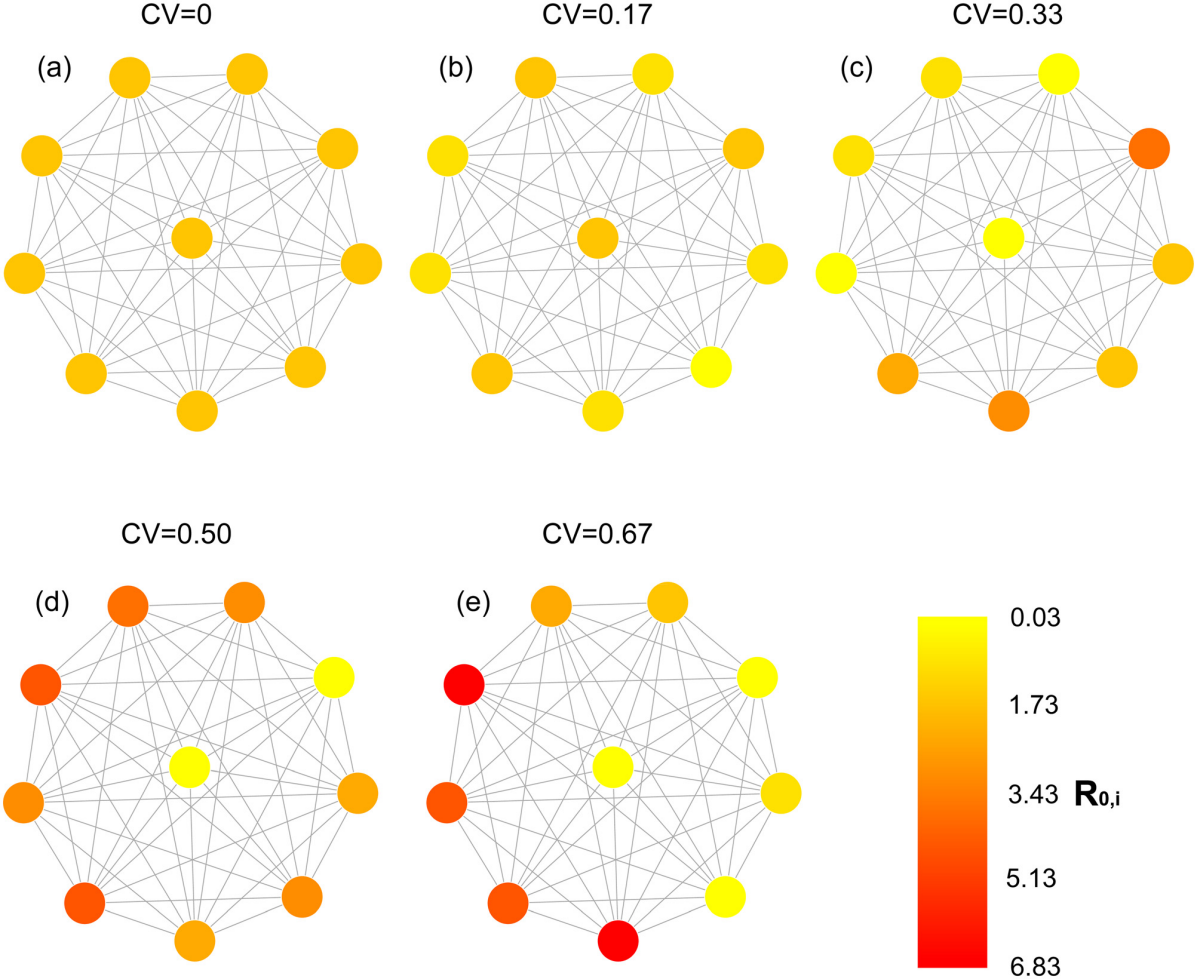
Network representation of simulated landscape configurations. Nodes represent patches characterized by their randomly generated *R*_0,*i*_, and links represent host movement. Each configuration represents a particular scenario of spatial heterogeneity in transmission intensity, which increases with increasing coefficient of variation (CV).

To study the implications of spatial heterogeneity in transmission intensity, in the presence of host movement, for disease prevalence and persistence, we generated a landscape composed of *Q* = 10 discrete patches connected by movement (Fig 1). We used this landscape to simulate a spatially homogeneous configuration in transmission intensity and four heterogeneous configurations (Fig 1). As with the two-patch analysis, the variation in transmission intensity was attained by varying the ratio of mosquitoes to humans *m*_*i*_, while keeping all other parameters constant (Table 1). The ratio of mosquitoes to humans in each patch was drawn from a normal distribution such that in the homogeneous configuration *m*_*i*_ = 60, and in the four heterogeneous configurations $m_{i}\overset{iid}{\sim }N(60,10)$, $m_{i}\overset{iid}{\sim }N(60,20)$, $m_{i}\overset{iid}{\sim }N(60,30)$, and $m_{i}\overset{iid}{\sim }N(60,40)$. This resulted in the same mean transmission intensity in each of the landscape configurations (${\bar{R}}_{0,i}$), although the range (min *R*_0,*i*_, max *R*_0,*i*_ varied among the five configurations: [2.17, 2.17], [1.04, 3.33], [0.03, 4.66], [0.03, 5.96], and [0.03, 6.83] from the homogeneous landscape to the most heterogeneous configuration, respectively (Fig 1). This resembles, in part, variation in malaria transmissibility reported in South America and Africa [1]. To determine how host movement affected persistence and prevalence, and how their relationship depended upon variation in patch transmissibility, we varied the rate of host movement between all patches (*k*) from 0 to 0.2 (days^−1^) in 1 × 10^−2^ increments. This rate was equal among all patches. Given that population size was also equal among patches we are evaluating the simple case where population size is constant and movement is symmetric among patches. We replicated this simulation 100 times for each configuration.

**Table 1:** Parameter values for patches in the simulated landscape. The ratio of mosquitoes to humans varied depending on landscape configuration where *s* = 0 for the homogeneous configuration and *s* = {0.17*m*, 0.33*m*, 0.5*m*, 0.67*m*} for the spatially heterogeneous configurations.

Parameter	Description	Value	Units	Reference(s)
*m*	Ratio of mosquitoes to humans	∼ *N*(60, *s*)	mosquitoes/human	
*a*	Mosquito biting rate	0.1	bites per mosquito per day	[49]
*b*	Effective transmission from mosquito to human	0.1	probability	[50]
*c*	Effective transmission from human to mosquito	0.214	probability	[51, 52]
*g*	Mosquito per-capita death rate	0.167	probability of mosquito dying per day	[53, 54]
*n*	Incubation period	10	days	[55, 56]
*r*	Recovery rate	0.0067	days^−1^	[57]
*N*	Total population size	9 × 10^6^	number of human hosts	
*k*	Rate of movement	[0, 0.2]	days^−1^	

## Results

### Two-patch analysis

To evaluate the effect of heterogeneity in transmission intensity on disease dynamics, we first proved analytically for the two-patch model that the network reproduction number *R*_0_, and the total disease prevalence lim_*t* → ∞_(*I*_1_(*t*)/*N*+*I*_2_(*t*)/*N*) increase with variance $V=\frac{1}{2}\left({\left(m_{1}-\bar{m}\right)}^{2}+{\left(m_{2}-\bar{m}\right)}^{2}\right)$, even if $\bar{m}=mean\{m_{1},m_{2}\}$, and consequently the average transmission intensity (*R*_01_+*R*_02_)/2 between the two regions, remains constant (see Theorems 0.0.2 and 0.0.4 in S3 Text). Because CV is proportional to the square root of the variance *V*, this implies that disease persistence and prevalence increase with CV. However, the influence of heterogeneity on *R*_0_ becomes less profound as connectivity between the two patches increases (see Proposition 0.0.3 in S3 Text).

### Multi-patch analysis

**Fig 2:**
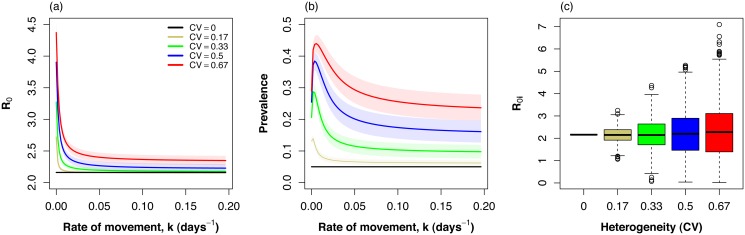
(a) The basic reproduction number *R*_0_ and (b) disease prevalence as a function of increasing movement rate (*k*) in a spatial network composed of 10 regions with varying levels of heterogeneity in transmission intensity. Lines represent means and shaded areas 95% confidence intervals. Spatial heterogeneity in transmission intensity increases with the coefficient of variation (CV). (c) Box-plots shows the distribution of patch-specific transmission intensities *R*_0,*i*_ in 100 simulations for each level of spatial heterogeneity. Note how variance increases with CV, while the average remains similar among configurations.

Spatial heterogeneity in transmission intensity increased long-term persistence of infection (*R*_0_) in the multi-patch system (Fig 2). Yet, increasing host movement-rate decreased *R*_0_ in the spatially heterogeneous scenarios. Spatial homogeneity resulted in the lowest *R*_0_ of all landscape configurations (Fig 2), which is consistent with our conclusions derived analytically from the two-patch system (see above). *R*_0_ in this homogeneous case was also independent of movement because the system was effectively a one patch system. In contrast, in all heterogeneous configurations, increasing host movement-rate resulted in a decrease in *R*_0_ that approached an asymptote. The value of this asymptote increased with increasing spatial heterogeneity (Fig 2), which is also consistent with our analytic results for the two-patch case.

**Fig 3:**
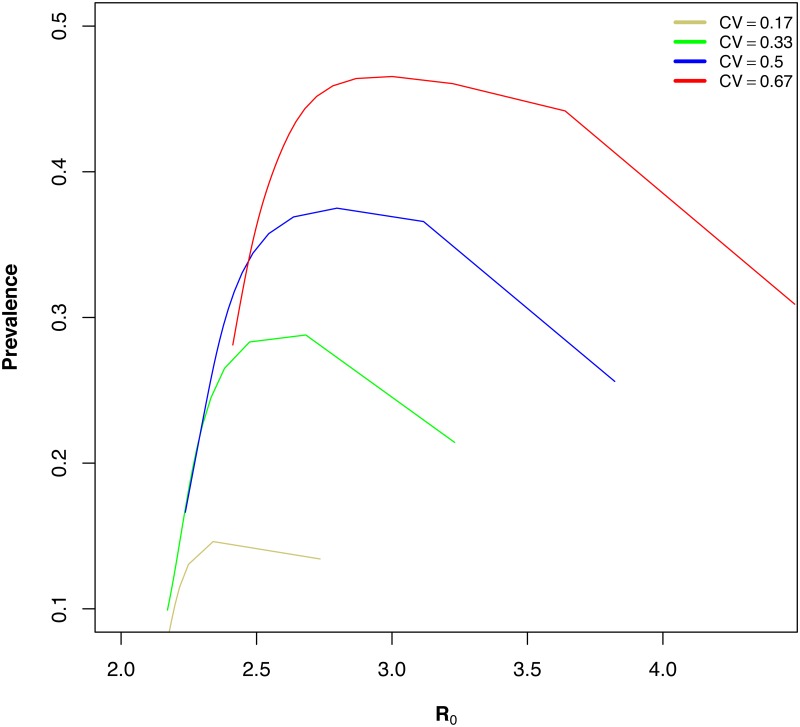
Non-monotonic relationship between *R*_0_ and prevalence. The figure shows four landscape configurations with spatial heterogeneity in transmission intensity for increasing rates of host movement.

Similarly, spatial heterogeneity in transmission intensity increased disease prevalence in the multi-patch system. Spatial homogeneity in transmission intensity resulted in the lowest prevalence of all landscape configurations (Fig 2). Maximum prevalence and the asymptotic prevalence with increasing spatial heterogeneity in transmission intensity, which again, agrees with our conclusions derived for the two-patch case. Disease prevalence initially increased with increasing movement, was maximized at relatively low movement rates and later decreased. The movement rate, *k*, that maximized prevalence increased with increasing heterogeneity and occurred at movement rates corresponding to once every 0.5 to 1.5 years. This suggests that the rate of movement required to maximize disease prevalence increases with increasing spatial heterogeneity in transmission intensity. Note that, in the simulations, mean *R*_0,*i*_ remained the same for all scenarios while variance increased with increasing coefficient of variation, as expected (Fig 2). In all heterogeneous configurations prevalence and *R*_0_ followed a non-monotonic relationship in the presence of host movement (Fig 3).

## Discussion

We have explored the way that disease prevalence and *R*_0_ — two important measures of mosquito-borne pathogen transmission — display a complex non-monotonic relationship as a result of spatial heterogeneity in mosquito density and human mobility. Heterogeneity in mosquito density and mosquito bionomic patterns affecting vectorial capacity drive spatially heterogeneous biting patterns, while human mobility connects isolated areas that can have very different mosquito populations. We illustrated these patterns analytically in a two-patch system, and numerically in a multi-patch extension of the Ross-Macdonald modeling framework. We showed that prevalence was maximized at low rates of movement, whereas *R*_0_ always decreased with increasing movement rates. These results suggest that the relationship between *R*_0_ and prevalence is intimately intertwined with the interaction between host movement and the degree of spatial heterogeneity in a region.

**Fig 4:**
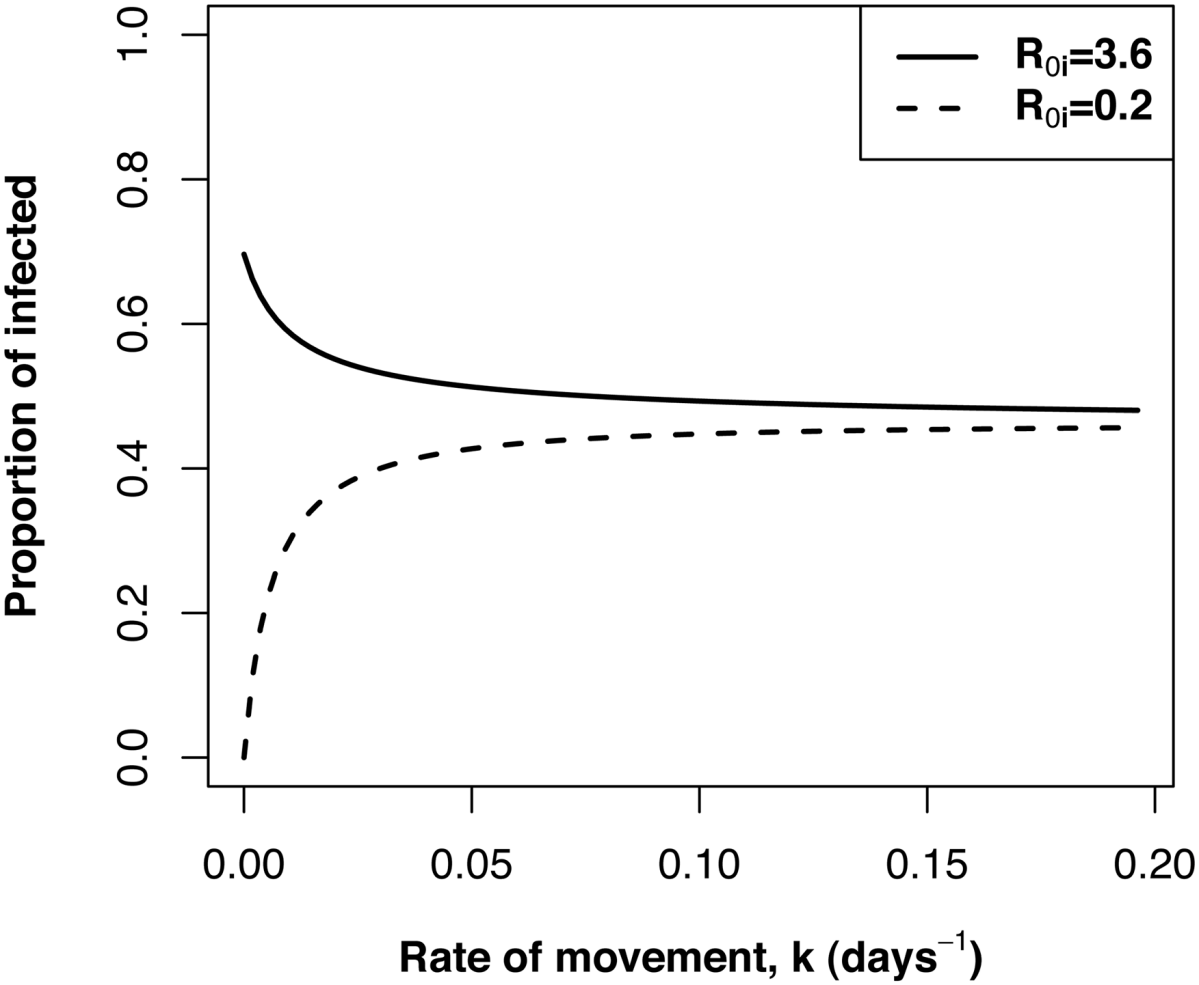
The change in the patch-specific proportion of infected hosts in a high transmission patch (*R*_0,*i*_ = 3.6) and a low transmission patch (*R*_0,*i*_ = 0.2) as a function of increasing rate of movement. The proportion of infected hosts in the low transmission patch increase with increasing rate of movement because it is receiving infected immigrants from other patches with high transmission. The proportion of infected hosts in the high transmission patch decrease with increasing rate of movement because of increasing emigration of infected hosts to other patches.

Transmission heterogeneity generally promotes persistence in host-parasite systems [18, 58–61]. This heterogeneity may have a spatial component arising from spatial variation in factors affecting mosquito ecology such as habitat distribution or host finding ability [25, 61]. Our results showed that disease persistence decreased with increasing rates of movement even in highly spatially heterogeneous landscapes with multiple transmission hotspots (Figs 1 and 2). At low rates of movement, transmission was highly heterogeneous, with high rates of transmission in some patches and low in others. *R*_0_ was higher in this scenario, because our calculation of *R*_0_ describes the average number of potential infections that arise from an average infected host in the system and thus its magnitude is being influenced by conditions in high transmission patches (Fig 4). Transmission becomes more homogeneous with increasing rate of movement resulting in individual patch transmissibility more similar to the overall average (Fig 4). A similar result was found in a study of the metapopulation dynamics of Schistosomiasis (bilharzia) [62], where increased social connectivity sometimes reduced large-scale disease persistence because as mobility increases infectious individuals spent less time in areas of high transmission distributing infection away from hotspots. Thus, acknowledging host movement patterns is required to better understand disease persistence in heterogeneous landscapes.

Results from our numerical simulations support previous theoretical and empirical work showing that disease prevalence is generally maximized at low to intermediate levels of movement [31, 63, 64]. Our results add to this body of theory by showing that the amount of movement required to achieve peak prevalence increases with increasing spatial transmission heterogeneity. At very low rates of movement, individuals spend most of their time in a single patch. In transmission hotspots most hosts are already infected at equilibrium and most bites do not yield new infections. A relatively small increase in movement will significantly increase the number of hosts exposed to very intense transmission (Fig 4). Therefore, as connectivity increases, the number of infectious bites in high transmission patches decrease, yet, this decrease is offset by the increase in the number of susceptibles that visit these patches. As connectivity continues to increase, hosts spend less time in high transmission patches resulting in a decrease in the number of hosts that become infected in high transmission patches. This causes the number of infectious bites in high transmission patches to decline, ultimately causing fewer people to be infected, and prevalence decreases. The different behaviors of prevalence and *R*_0_ in the presence of spatial heterogeneity and mobility suggest a role for models including mobility and spatial scale in the estimation of prevalence based on *R*_0_ estimates, because the assumed positive relationship between the two is disrupted [21].

Reproduction numbers (*R*_0_) are useful to understand the intensity of transmission in a region and are often used to design and evaluate control measures of mosquito-borne diseases. The estimation of *R*_0_ can be done using several different methods, including estimating number of infectious bites on a person per year [1, 61, 65, 66]. Generally, depending on the assumptions about superinfections and density dependence among parasites, *R*_0_ is proportional to the inverse of the fraction of uninfected individuals at equilibrium (i.e. *R*_0_ and prevalence are positively correlated) [67, 68]. Yet, this relationship between prevalence and *R*_0_ has been shown to be disrupted by heterogeneous biting [18, 58, 61, 67–69]. Our analysis of the two-patch system illustrated that increasing heterogeneity increases both prevalence and *R*_0_, but the multi-patch numerical simulations show this effect is diminished as connectivity increases suggesting that the human“activity space” — or how humans spend time between areas of varying mosquito densities — is also an important determinant of the relationship between *R*_0_ and prevalence [70]. For example, assuming that transmission intensity across two regions is the average of the transmission intensity in each region will underestimate the disease burden, particularly at low to intermediate levels of connectivity. Therefore our results emphasize the necessity for reasonable estimates of host movement rates, because individual patch transmission intensities do not uniquely determine overall transmission intensity and prevalence.

Our findings have important practical implications for mosquito-borne disease control in heterogeneous landscapes in the presence of symmetric host movement. Our results show that the dynamics of spatially heterogeneous system are driven primarily by the characteristics of areas with the highest potential for transmission by mosquitoes, which supports the idea that hotspots should be targeted for control efforts. If control strategies are untargeted these high transmission areas may represent residual areas where the disease persists with the potential to re-colonize others [32, 71, 72], or maintain transmission throughout the system. This is shown by the persistence of malaria in many landscape scenarios, despite *R*_0,*i*_ < 1 in many patches (Fig 2a and 2c). Thus, controlling malaria transmission in areas with heterogeneous transmission requires a combination of interventions that include mosquito control, the reduction of human infectious reservoirs, and vaccination targeted towards high transmission areas [32].

Finally, human movement between areas often changes over time, and predicting how these changes will affect transmission and prevalence requires understanding the effect of connectivity on prevalence and the initial degree of movement. If human movement is very low initially, an increase in movement is likely to increase endemic prevalence, while an initially high human movement will likely result in a decrease in endemicity if movement increases further. Therefore, knowing the degree of connectivity between areas and how connectivity changes over time is also important to management and elimination planning [32]. Recent studies are beginning to analyze human movement in relation to mosquito-borne pathogen transmission [70, 73–75], and these show great promise for improving models of mosquito-borne pathogen transmission across geographic scales.

## Supporting Information

S1 Text Multi-patch model derivation. Derivation of a multi-patch extension of the Ross-Macdonald model in Eq (1) from a single-patch model.
(PDF)

S2 Text Theorem 0.0.1. Mathematical proof showing that system of equations in (1) exhibit *uniform weak persistence*.
(PDF)

S3 Text Theorem 0.0.2, Proposition 0.03 and Theorem 0.0.4. Mathematical proofs showing that total equilibrium prevalence in a two-patch system is an increasing function of the variance in transmission intensity.
(PDF)
