# Supplementary material for: Spatial Heterogeneity, Host Movement and Mosquito-Borne Disease Transmission
Source: PLoS One. 2015 Jun 1;10(6):e0127552. doi: 10.1371/journal.pone.0127552 (PMC4452543; doi:10.1371/journal.pone.0127552)
Supplement: S1 Text — Derivation of a multi-patch extension of the Ross-Macdonald model in Eq (1) from a single-patch model. (PDF) [file pone.0127552.s001.pdf]

Spatial heterogeneity, host movement and vector-borne disease transmission

Miguel A. Acevedo[1,*], Olivia Prosper[2], Kenneth Lopiano[3], Nick Ruktanonchai[4], T. Trevor Caughlin[4], Maia Martcheva[5], Craig W. Osenberg[4], David L. Smith[6].

1 University of Puerto Rico–Río Piedras, Department of Biology, San Juan, PR, USA

2 Dartmouth College, Department of Mathematics, Hanover, NH, USA

3 Statistical and Applied Mathematical Sciences Institute, Research Triangle Park, NC, USA

4 University of Florida, Department of Biology, Gainesville, FL, USA

5 University of Florida, Department of Mathematics, Gainesville, FL, USA

6 Department of Epidemiology and Malaria Research Institute, John Hopkins Bloomberg School of Public Health, Baltimore, MD, USA

∗ **E-mail: miguel.acevedo7@upr.edu**

Supporting Information 1

The Ross-Macdonald model describes the proportion of humans infected (i) and the proportion of mosquitoes infected (z) over time:

$$\frac{dz}{dt} = aci(1-z) - gz \tag{1}$$

$$\frac{di}{dt} = mabz(1-i) - ri, \tag{2}$$

where $1-z$ and $1-i$ describe the proportion of susceptible mosquitoes and susceptible humans respectively.

We modified the Ross-Macdonald model [1] to account for the change in the number of humans infected (instead of the proportion), leading to the following set of equations:

$$\frac{dz}{dt} = ac\frac{I}{N}(1-z) - gz \tag{3}$$

$$\frac{dI}{dt} = mabz(N-I) - rI, \tag{4}$$

where N is the total size of the human population of interest, and I is the number of humans infected with malaria. Thus, $N - I$ represents the number of humans who are susceptible.

The parameter a is the rate mosquitoes bite humans, c is the probability a mosquito becomes infected given that the mosquito has bitten an infected human, b is the probability a susceptible human being was infected given a mosquito bite from an infected mosquito, g is the mosquito death rate, m is the ratio of the abundance of mosquitoes to humans and r is the recovery rate of infected humans. In this model there is no immunity after infection. Because the model without human demography is asymptotically equivalent to the model with demography, and because we are interested in equilibrium behavior, we omitted human births and deaths for simplicity.

We have incorporated two modifications to the original model. First, given the high mortality rate of mosquitoes, many may not survive to become infectious; hence we replaced $(1 - z)$ with $e^{-gn} - z$ [2], where n is the extrinsic incubation period.

Second, we incorporated immigration and emigration between all Q patches resulting in the following system of $3 \times Q$ equations:

$$\frac{dz_i}{dt} = a_i c_i \frac{I_i}{N_i}(e^{-g_i n_i} - z_i) - g_i z_i \tag{5}$$

$$\frac{dI_i}{dt} = m_i a_i b_i z_i (N_i - I_i) - r_i I_i - I_i \sum_{j \neq i}^{Q} k_{ji} + \sum_{j \neq i}^{Q} k_{ij} I_j \tag{6}$$

$$\frac{dN_i}{dt} = -N_i \sum_{j \neq i}^{Q} k_{ji} + \sum_{j \neq i}^{Q} k_{ij} N_j \tag{7}$$

Under the assumptions specified in the main manuscript, our model simplifies to:

$$\begin{aligned} \frac{dz_i}{dt} &= ac\frac{I_i}{N}(e^{-gn} - z_i) - gz_i \\ \frac{dI_i}{dt} &= m_i abz_i(N - I_i) - rI_i - I_i \sum_{j \neq i}^{Q} k + \sum_{j \neq i}^{Q} kI_j \end{aligned} \tag{8}$$

References

1. Bailey NTJ (1982) The biomathematics of malaria. Oxford University Press.

2. Smith D, McKenzie F (2004) Statics and dynamics of malaria infection in *Anopheles* mosquitoes.

Malaria Journal 3: 13.
