# Supplementary material for: Spatial Heterogeneity, Host Movement and Mosquito-Borne Disease Transmission
Source: PLoS One. 2015 Jun 1;10(6):e0127552. doi: 10.1371/journal.pone.0127552 (PMC4452543; doi:10.1371/journal.pone.0127552)
Supplement: S2 Text — Mathematical proof showing that system of equations in (1) exhibit uniform weak persistence. (PDF) [file pone.0127552.s002.pdf]

Spatial heterogeneity, host movement and vector-borne disease transmission

Miguel A. Acevedo[1,*], Olivia Prosper[2], Kenneth Lopiano[3], Nick Ruktanonchai[4], T. Trevor Caughlin[4], Maia Martcheva[5], Craig W. Osenberg[4], David L. Smith[6].

1 University of Puerto Rico–Río Piedras, Department of Biology, San Juan, PR, USA

2 Dartmouth College, Department of Mathematics, Hanover, NH, USA

3 Statistical and Applied Mathematical Sciences Institute, Research Triangle Park, NC, USA

4 University of Florida, Department of Biology, Gainesville, FL, USA

5 University of Florida, Department of Mathematics, Gainesville, FL, USA

6 Department of Epidemiology and Malaria Research Institute, John Hopkins Bloomberg School of Public Health, Baltimore, MD, USA

∗ **E-mail: miguel.acevedo7@upr.edu**

Supporting Information 2

Theorem 0.0.1. *If $R_0 > 1$, System (1) in the main text exhibits uniform weak persistence; that is, there exists an $\epsilon > 0$ such that*

$$\limsup_{t\to\infty} \sum_{i=1}^{Q} I_i(t) + z_i(t) \geq \epsilon,$$

whenever $\sum_{i=1}^{Q} I_i(0) + z_i(0) > 0$.

Proof. By way of contradiction, suppose $\limsup_{t\to\infty} \sum_{i=1}^{Q} I_i(t) + z_i(t) < \epsilon$ for all $\epsilon > 0$. Then, $I_i(t) \leq \epsilon$ and $z_i(t) \leq \epsilon$ for all t, and for each $i = 1, 2, \ldots, Q$. From System 1 in the main text, we obtain the following inequalities:

$$\frac{dI_i(t)}{dt} \geq \xi_i(\epsilon) z_i - [r + (Q-1)k] I_i + k \sum_{j \neq i}^{Q} I_j$$
$$\frac{dz_i(t)}{dt} \geq \eta_i(\epsilon) I_i - g z_i, \quad i = 1, \ldots, Q$$

where $\xi_i(\epsilon) = m_i ab(N-\epsilon)$ and $\eta_i(\epsilon) = ac\frac{I_i}{N}(e^{-gn}-\epsilon)$. Note that

$$\frac{dX_i(t)}{dt} = \xi_i(\epsilon)y_i - [r+(Q-1)k]X_i + k\sum_{j\neq i}^{Q} X_j$$
$$\frac{dy_i(t)}{dt} = \eta_i(\epsilon)X_i - gy_i, \quad i=1,\ldots,Q$$

is a linear system of $2Q$ equations, and can be written in the form $\mathbf{W}' = J(\epsilon)\mathbf{W}$, where

$$\mathbf{W} = (y_1, y_2, \ldots, y_Q, X_1, X_2, \ldots, X_Q)^T,$$

and

$$J(\epsilon) = \begin{bmatrix} J_{1,1} & J_{1,2}(\epsilon) \\ J_{2,1}(\epsilon) & J_{2,2} \end{bmatrix},$$

where each $J_{i,j}$ is a $Q\times Q$ block matrix defined by $J_{1,1} = diag(-g,-g,\ldots,-g)$, $J_{1,2}(\epsilon) = diag(\eta_1(\epsilon),\eta_2(\epsilon),\ldots,\eta_Q(\epsilon))$, $J_{2,1}(\epsilon) = diag(\xi_1(\epsilon),\xi_2(\epsilon),\ldots,\xi_Q(\epsilon))$, and

$$J_{2,2}(\epsilon) = \begin{bmatrix} -[r+(Q-1)k] & k & \cdots & k \\ k & -[r+(Q-1)k] & \cdots & k \\ \vdots & \vdots & \ddots & \vdots \\ k & k & \cdots & -[r+(Q-1)k] \end{bmatrix}.$$

Because $\xi_i(0) = m_i abN = \alpha_i N$ and $\eta_i(0) = \dfrac{ace^{-gn}}{N} = \dfrac{\beta}{N}$, $J(0)$ is precisely the Jacobian of System (1) in the main text evaluated at the disease-free equilibrium. Furthermore, $I_i(t) \geq X_i(t)$ for all t and for each i, provided they have the same initial conditions.

Let $F(\epsilon)$ and V be such that $F(\epsilon) = \begin{bmatrix} 0 & J_{1,2}(\epsilon) \\ J_{2,1}(\epsilon) & 0 \end{bmatrix}$, and $V = \begin{bmatrix} J_{1,1} & 0 \\ 0 & J_{2,2} \end{bmatrix}$. Then, $J(\epsilon) = F(\epsilon) - V$.

Let $R(\epsilon) := (\rho(F(\epsilon)V^{-1}))^2$, the square of the spectral radius of the matrix FV^{-1}. Then, $\lim_{\epsilon\to 0} R(\epsilon) = R_0$. Because $R_0 > 1$, this implies that there exists an $\epsilon' > 0$ such that $R(\epsilon') > 1$. Because $F(\epsilon')$ is nonnegative and V is a non-singular M-matrix, $\rho(F(\epsilon')V^{-1}) > 1$ implies that at least one eigenvalue lies in the

right half of the complex plane. Hence, the spectrum of $J(\epsilon')$ has an eigenvalue with positive real part, implying that $\lim_{t\to\infty} I_i(t) = \infty$ or $\lim_{t\to\infty} z_i(t) = \infty$ for some i, which is a contradiction. Therefore, the conclusion of the theorem holds.

$\square$
