# Supplementary material for: Spatial Heterogeneity, Host Movement and Mosquito-Borne Disease Transmission
Source: PLoS One. 2015 Jun 1;10(6):e0127552. doi: 10.1371/journal.pone.0127552 (PMC4452543; doi:10.1371/journal.pone.0127552)
Supplement: S3 Text — Mathematical proofs showing that total equilibrium prevalence in a two-patch system is an increasing function of the variance in transmission intensity. (PDF) [file pone.0127552.s003.pdf]

Spatial heterogeneity, host movement and vector-borne disease transmission

Miguel A. Acevedo[1,*], Olivia Prosper[2], Kenneth Lopiano[3], Nick Ruktanonchai[4], T. Trevor Caughlin[4], Maia Martcheva[5], Craig W. Osenberg[4], David L. Smith[6].

1 University of Puerto Rico–Río Piedras, Department of Biology, San Juan, PR, USA

2 Dartmouth College, Department of Mathematics, Hanover, NH, USA

3 Statistical and Applied Mathematical Sciences Institute, Research Triangle Park, NC, USA

4 University of Florida, Department of Biology, Gainesville, FL, USA

5 University of Florida, Department of Mathematics, Gainesville, FL, USA

6 Department of Epidemiology and Malaria Research Institute, John Hopkins Bloomberg School of Public Health, Baltimore, MD, USA

∗ **E-mail: miguel.acevedo7@upr.edu**

Supporting Information 3

Two-patch analysis

We first made the following assumptions:

1. Each patch has identical parameters, with the exception of the ratio of mosquitoes to humans m_1 and m_2.

2. $\bar{m} := \frac{m_1+m_2}{2}$, the average of m_1 and m_2, is fixed.

3. $\bar{\alpha} := \frac{m_1}{m_2}$, where, without loss of generality, $m_1 > m_2$ so that $\alpha \in (1, \infty)$.

Theorem 0.0.1. *Under the above assumptions, R_0 is an increasing function of the variance*

$$V = \frac{(m_1 - \bar{m})^2 + (m_2 - \bar{m})^2}{2}.$$

Proof. Note that $\dfrac{\partial R_0}{\partial V} = \dfrac{\partial \bar{\alpha}}{\partial V} \cdot \dfrac{\partial R_0}{\partial \bar{\alpha}}$. We will first show that $\dfrac{\partial R_0}{\partial \bar{\alpha}} > 0$.

Assumptions 2 and 3 imply that

$$(m_1, m_2) = \left(\frac{2\bar{\alpha}\bar{m}}{\bar{\alpha}+1}, \frac{2\bar{m}}{\bar{\alpha}+1} \right). \tag{1}$$

Using the definition of R_0 described in the previous section, it is straightforward to show that R_0 for our two-patch system is a special case of the R_0 derived in [1]. In [1],

$$R_0 = \frac{1}{2\sigma}\left(s_1t_2 + s_2t_1 + \sqrt{(s_1t_2+s_2t_1)^2 - 4s_1s_2\sigma}\right),$$

where $\sigma = k_{12}r_1 + k_{21}r_2 + r_1r_2$, $s_i = \frac{\alpha_i\beta_i}{g_i}$, and $t_i = r_i + k_{ji}$. Since all patch parameters, except for m_1 and m_2 are identical in this manuscript, we take $k = k_{12} = k_{21}$, $r = r_1 = r_2$, $\beta = \beta_1 = \beta_2$, and $g = g_1 = g_2$. Subsequently, we have $\sigma = 2kr + r^2$, $s_i = \frac{\alpha_i\beta}{g}$, and $t = r + k = t_1 = t_2$.

Note that $s_1t_2 + s_2t_1 = s_2t_2\left(\frac{s_1}{s_2} + \frac{t_1}{t_2}\right) = s_2t_2(\bar{\alpha}+1)$. So, $R_0 = \frac{s_2t}{2\sigma}\left(\bar{\alpha}+1+\sqrt{(\bar{\alpha}+1)^2 - 4\bar{\alpha}\frac{\sigma}{t^2}}\right)$.

Recall that $s_2 = m_2\eta$, where $\eta = a^2bce^{-gn}/g$ (under the simplifying parameter assumptions). From the expression for m_2, we obtain $s_2 = \frac{2\eta\bar{m}}{\bar{\alpha}+1}$, which yields (after simplification) an expression for R_0 as a function of $\bar{\alpha}$:

$$R_0(\bar{\alpha}) = \eta\bar{m}\frac{t}{\sigma}\left(1 + \sqrt{1 - 4\frac{\bar{\alpha}}{(\bar{\alpha}+1)^2}\cdot\frac{\sigma}{t^2}}\right).$$

Now, it remains to show that $\frac{\partial R_0}{\partial\bar{\alpha}} > 0$ on $(1,\infty)$. Only the argument of the square root in R_0 depends on $\bar{\alpha}$. Thus, to determine the sign of $\frac{\partial R_0}{\partial\bar{\alpha}}$, we first note that $\frac{\partial}{\partial\bar{\alpha}}\left(\frac{\bar{\alpha}}{(\bar{\alpha}+1)^2}\right) = \frac{1-\bar{\alpha}}{(\bar{\alpha}+1)^3} < 0$ on $(1,\infty)$. From this, it is clear that R_0 is an increasing function of $\bar{\alpha}$.

We conclude the proof by writing V as a function of $\bar{\alpha}$, and illustrating that $\frac{\partial\bar{\alpha}}{\partial V}$ is also positive. Substituting Equation (1) into the expression for the two-patch variance V, we find that $V(\bar{\alpha}+1)^2 = \bar{m}^2(\bar{\alpha}-1)^2$. Implicit differentiation with respect to V, and treating $\bar{\alpha}$ as a function of V, yields:

$$\frac{\partial\bar{\alpha}}{\partial V} = \frac{(\bar{\alpha}+1)^3}{4\bar{m}^2(\bar{\alpha}-1)},$$

which is positive. In the above calculation, we used the fact that $V(\bar{\alpha}+1)^2 = \bar{m}^2(\bar{\alpha}-1)^2$ to write the expression in terms of only $\bar{m}$ and $\bar{\alpha}$. Consequently, R_0 is an increasing function of V.

□

Proposition 0.0.2. $\frac{\partial}{\partial k}\frac{\partial R_0}{\partial\bar{\alpha}} < 0$.

Proof. Calculating $R_0'(\bar{\alpha})$ explicitly, we obtain: $R_0'(\bar{\alpha}) = 2\eta\bar{m}\left(1 - 4\frac{\bar{\alpha}}{(\bar{\alpha}+1)^2}\cdot\frac{\sigma}{t^2}\right)^{-\frac{1}{2}} \cdot \frac{\bar{\alpha}-1}{(\bar{\alpha}+1)^3}\cdot\frac{1}{t}$. Clearly, $\frac{\partial}{\partial k}\left(\frac{1}{t}\right) < 0$ since $t = r + k$, and $\frac{\partial}{\partial k}\left(\frac{\sigma}{t^2}\right) = -\frac{2rk}{(r+k)^3} < 0$. Since $1/t$ and σ/t^2 are both decreasing functions of k and no other terms in $\frac{\partial R_0}{\partial \bar{\alpha}}$ depend on k, we observe that $\frac{\partial R_0}{\partial \bar{\alpha}}$ must decrease with k.

□

Theorem 0.0.3. *The total equilibrium prevalence in the two-patch system, $I^* = I_1^* + I_2^*$ is an increasing function of the variance V.*

Proof. The equilibrium equations for our two-patch system are

$$0 = ac\frac{I_i}{N}(e^{-gn} - z_i) - gz_i, \quad i = 1,2$$
$$0 = m_i abz_i(N - I_i) - rI_i - kI_i + kI_j, \quad i = 1,2$$

Solving for z_i in the first equation and substituting this quantity into the second equation, we obtain the equilibrium equations

$$0 = \frac{m_i a^2 bce^{-gn}}{acI_i + gN}(N - I_i) - (r+k)I_i + kI_j, \quad i = 1,2,$$

which is a special case of the equilibrium equations in [1].

From equations (33)-(34) in [1],

$$\frac{\partial I_1^*}{\partial \alpha_1} = -\frac{C_{\alpha_1}A_2}{A_1A_2 - B_1B_2} \tag{2}$$
$$\frac{\partial I_2^*}{\partial \alpha_1} = \frac{C_{\alpha_1}B_2}{A_1A_2 - B_1B_2}, \tag{3}$$

where

$$\begin{aligned}
A_i &= \alpha_i\beta(N_i^* - 2I_i^*) - t(2\beta I_i^* + gN_i^*) + k\beta I_j^* \\
&= \alpha_i\beta(N - 2I_i^*) - t(2\beta I_i^* + gN) + k\beta I_j^* \\
B_i &= k(\beta I_i^* + gN_i^*) \\
&= k(\beta I_i^* + gN) \\
C_{\alpha_1} &= \beta I_1^*(N_1^* - I_1^*) \\
&= \beta I_1^*(N - I_1^*)
\end{aligned}$$

Recall that $\alpha_1 = m_1 abe^{-gn} = \frac{2\bar{\alpha}\bar{m}}{\bar{\alpha}+1} abe^{-gn}$.

This fact, along with equations (2)-(3), implies that

$$\frac{\partial I^*}{\partial\bar{\alpha}} = \frac{\partial\alpha_1}{\bar{\alpha}}\frac{\partial I^*}{\partial\alpha_1} = \frac{2\bar{m}abe^{-gn}}{(\bar{\alpha}+1)^2} \cdot \frac{C_{\alpha_1}(B_2 - A_2)}{A_1A_2 - B_1B_2}.$$

Proposition 5.0.1 in [1] states that $A_1A_2 - B_1B_2 > 0$, and the proof of this proposition states that $A_2 < 0$. Thus, $B_2 - A_2 > 0$ implies that $\frac{\partial I^*}{\partial\bar{\alpha}} > 0$. Recall that in the proof of the previous theorem, we showed that $\partial\bar{\alpha}/\partial V > 0$; consequently, I^* is an increasing function of the variance V.

□

References

1. Prosper O, Ruktanonchai N, Martcheva M (2012) Assessing the role of spatial heterogeneity and human movement in malaria dynamics and control. Journal of Theoretical Biology 303: 1–14.
